# BioGeomancer: Automated Georeferencing to Map the World's Biodiversity Data

**DOI:** 10.1371/journal.pbio.0040381

**Published:** 2006-11-14

**Authors:** Robert P Guralnick, John Wieczorek, Reed Beaman, Robert J Hijmans, the BioGeomancer Working Group

## Abstract

The BioGeomancer Project provides a toolkit to georeference data and specimens collected for natural history collections, a crucial task if the potential of these specimens is to be fully realized.

Over the past 250 years, biologists who were interested in describing and understanding patterns of biological diversity have gone into the field to observe and collect species. Conservation of the specimens and data collected through these explorations has produced an irreplaceable archive of life on Earth [1]. Today, the billions of specimens in natural history collections, such as dried plants and stuffed birds, play a fundamental role in generating new knowledge about biodiversity and in guiding its conservation. Yet, the potential of this vast store of data is much greater than is currently realized.

There are three main challenges to the effective use of specimens in natural history collections and of related observation data, such as bird counts. The first challenge is to capture the information associated with specimens, such as species name and the day and locality of collection, in computer databases. The name of an organism and the location in which it occurs are information elements most widely used by consumers of biodiversity data—including researchers, educators, natural resource managers, and the general public. The second challenge is to make this information, housed in many hundreds of separate institutes, easily accessible via the Internet. Through a large number of digitization and computer application development efforts, these challenges have been partially overcome, as is illustrated by the nearly 100 million species-occurrence records that are currently available through the Global Biodiversity Information Facility (GBIF; http://www.gbif.net). Many of these records have never before been electronically accessible, let alone from a single point of access that uses sophisticated protocols to directly retrieve information from primary data custodians. The third grand challenge, and the focus of this article, is to further increase the value of these species-occurrence records by converting the textual descriptions of places where data and specimens were collected (locality descriptions such as “North Beach, Point Reyes, Marin County, California”) into their corresponding geographic coordinates—to georeference them.

The number of species-occurrence records worldwide that are not currently georeferenced is astounding; more than 99% of the one billion or more biological specimen records [2,3,4] lack geographic coordinates. This severely limits the degree to which past and current distributions of species can be mapped and analyzed in combination with spatial data from other disciplines (e.g., climatology, geology, geography, social sciences) [5].

Georeferencing is time consuming, especially if it is carried out on a specimen-by-specimen basis. Each georeference often takes as much time as digitizing the entire remainder of the occurrence record. At this level of effort, data managers of most natural history collections cannot afford to georeference species-occurrence data. Because so many records remain without geographic coordinates, individual researchers are forced to georeference the localities in the records they use, usually without access to the primary information such as field notes. The coordinates assigned to localities are typically not returned to the data custodians, leading to huge efficiency losses across the research and conservation system. Moreover, the data are often of poor quality, unstandardized and undocumented due to limited understanding of the concepts of georeferencing and spatial location and to a lack of access to specialized geographic information system (GIS) tools. Empirical evidence suggests that a large fraction of available georeferences are in error [6], yet formal methods for checking for errors are rarely applied.

In summary, the current situation for georeferencing is characterized by the following major shortcomings: (i) The process is slow—an average of several minutes per locality. (ii) With a few exceptions, the accuracy and precision of the assigned coordinates is unknown. (iii) A large fraction of available coordinates are demonstrably inconsistent with the rest of the locality information. (iv) The materials and methods used are poorly documented. (v) Many localities are georeferenced many times over—but not likely with the same results.

The BioGeomancer Project is an international collaborative effort to build a Web-based automated georeferencing toolkit that addresses all of the shortcomings listed above. The BioGeomancer toolkit is being developed by a coalition that includes individuals with expertise in manual and semi-automated georeferencing, GIS, collection management, software engineering, protocol standards, and data standards. The toolkit provides dramatic improvements over existing common practice by: (i) increasing the rate at which locations can be georeferenced (at least 5-fold) by focusing on automated methods and batch processing; (ii) estimating the uncertainty associated with the coordinates of each record; (iii) testing for errors and assuring consistency; (iv) enhancing the value of the data by defining and applying documented data standards; and (v) providing essential information about the data processing steps so that the results can be tested, replicated, and easily improved upon. By creating freely available georeferencing tools backed by high-quality digital geospatial data, BioGeomancer removes the cost of acquiring these resources at individual institutions and provides a standard mechanism for accomplishing a core biodiversity data management task.

BioGeomancer provides a set of tools that are necessary and sufficient to complete all the tasks that would otherwise be accomplished manually (see Figure 1 for a simple flow diagram showing how BioGeomancer works). One core capability of BioGeomancer is natural language processing—the interpretation of textual locality descriptions into their semantic components. After records containing locality information are uploaded to the website, one or more methods for natural language processing parse parts of a locality description into data fields required for spatial interpretation—components such as place name(s), offset(s), units, and direction.

**Figure 1:**
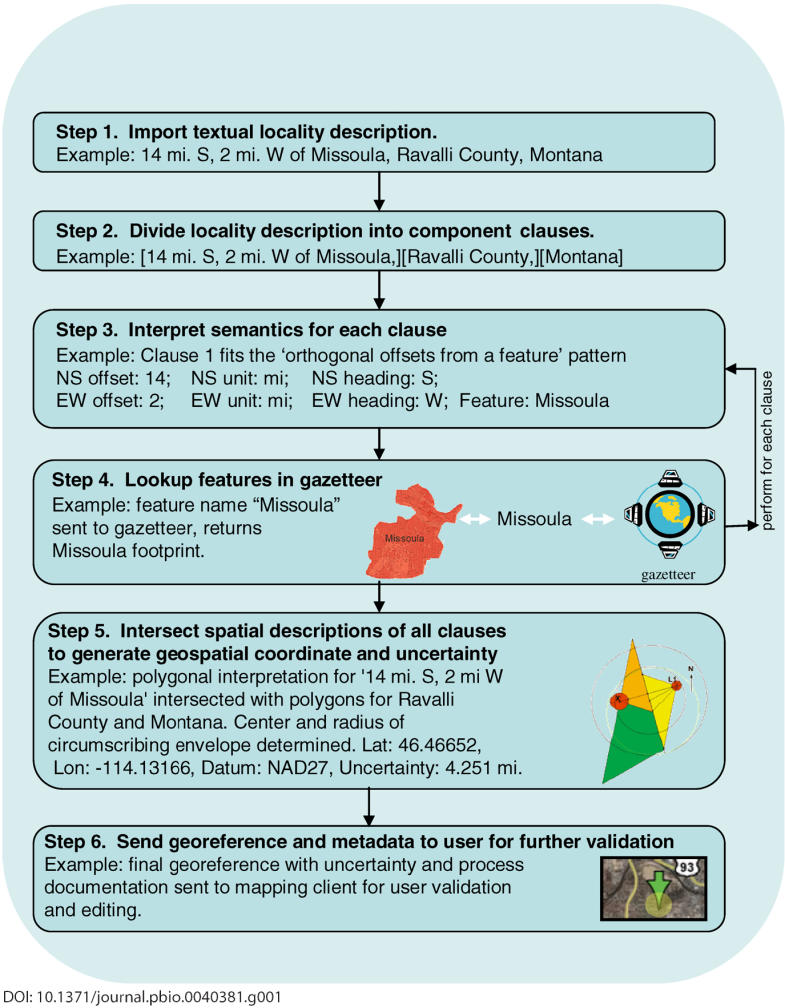
Diagram showing how BioGeomancer converts a textual locality description into a geospatial description suitable for use in geographic information systems.

Successfully parsed locality descriptions are georeferenced by looking up the named place(s) in a gazetteer and using the information found there along with offsets and the semantics of the locality. The program estimates the spatial uncertainty of a georeference [7], taking into account the ambiguities of the original description and the quality of the spatial data sources. The results can be checked for consistency, mapped for visual inspection, spatially and textually edited, and downloaded, all with tools available in the application. Validation tools are also available that help flag outlier records that might represent errors in input data or their interpretation.

BioGeomancer initially supports the interpretation of localities in English, Spanish, and Portuguese, although in the future, we hope to add capabilities for other languages by further developing the language-specific component needed to interpret locality descriptions. BioGeomancer is accessible at http://www.biogeomancer.org.
